# Clinicopathological, prognostic and predictive value of CD166 expression in colorectal cancer: a meta-analysis

**DOI:** 10.18632/oncotarget.17442

**Published:** 2017-04-26

**Authors:** Susu Han, Wei Yang, Shaoqi Zong, Hongjia Li, Shanshan Liu, Wen Li, Qi Shi, Fenggang Hou

**Affiliations:** ^1^ Oncology Department of Shanghai Municipal Hospital of Traditional Chinese Medicine, Shanghai TCM University, Shanghai, People's Republic of China

**Keywords:** CD166, expression, CRC, survival, clinical features

## Abstract

CD166 has been identified as an important cancer stem cell (CSC) marker in colorectal cancer (CRC). The purpose of our study was to investigate the relationship between CD166 expression and clinical features and to examine the role of CD166 expression on the survival of patients with CRC. A total of 15 studies with 3,332 cases were identified in this meta-analysis. The pooled OR indicated that CD166 expression was significantly higher in CRC than in colonic adenomas or normal colonic mucosa (OR = 3.48, *P* = 0.002 and OR = 55.13, *P* = 0.017, respectively). CD166 expression was found to be negatively correlated with vascular invasion (OR = 0.75, *P* = 0.017), but it was not associated with gender, tumor location, lymph node status, distant metastasis, clinical stage, T classification or tumor differentiation. Meanwhile, CD166 expression was not associated with the prognosis of overall survival (OS) (HR = 1.20, 95% CI = 0.45-3.22, *P* = 0.72) in multivariate regression analysis. One study reported that CD166 expression may be a predictor of survival in stage II CRC patients using multivariate logistic regression analysis (OS: OR = 9.97, *P* = 0.035; disease-specific survival: OR = 29.02, *P* = 0.011). Our findings suggest that CD166 expression may be correlated with CRC carcinogenesis and a decreased risk of vascular invasion, and it may become a predictive biomarker of survival for stage II CRC patients, but additional studies with large sample sizes are essential to validate the prognostic and predictive values of CD166 expression.

## INTRODUCTION

Worldwide, colorectal cancer (CRC) is one of the most frequent malignant tumors and the fourth leading cause of cancer-related deaths in human cancers. Based on global cancer statistics, approximately 1,360,600 new cases of CRC were clinically diagnosed, and CRC caused an estimated 693,900 deaths, in 2012 [1]. Although the therapeutic opportunities have markedly improved in recent years for CRC patients, patients with liver, lung or lymph node metastases (advanced stage) still have a poor prognosis, with a 5-year survival rate of ∼10% [2, 3].

Increasing evidence suggests that a small subpopulation of cancer cells, termed cancer stem cells (CSCs), are associated with self-renewal and uncontrolled proliferation, differentiation, and tumorigenicity [4, 5]. Some studies have shown that CSCs are responsible for the carcinogenesis, progression, metastasis and prognosis of human cancers [6–8]. Some CSC expression markers have become prognostic biomarkers in CRC (i.e., CD133, ALDH1 and Lgr5) [9–11]. CD166 is an important CSC marker; it is located on human chromosome 3q13.11, and it is also called activated leukocyte cell adhesion molecule (ALCAM) [12]. CD166 is involved in many biological functions, including CD166-CD6/CD166 cell-cell interactions, T-cell stimulation and proliferation, angiogenesis, and hematopoiesis [13–15]. CD166 expression is closely correlated with various types of human cancers, including CRC [16–18].

There are some inconsistent and controversial conclusions about CD166 expression in CRC. For example, a significant correlation between CD166 expression and overall survival (OS) of patients was reported based on multivariate analysis by Weichert *et al* [19]. However, Ribeiro *et al* reported that CD166 expression was not related to OS of patients with CRC using multivariate analysis [20]. Thus, we evaluated the prognostic and predictive role of CD166 expression in CRC patients with multivariate analysis. Moreover, we also evaluated the associations of CD166 expression between CRC and colonic adenomas and between CRC and normal colonic mucosa. Finally, we analyzed the correlation of CD166 expression with clinicopathological characteristics in this study.

## RESULTS

### Characteristics of relevant studies

**Figure 1:**
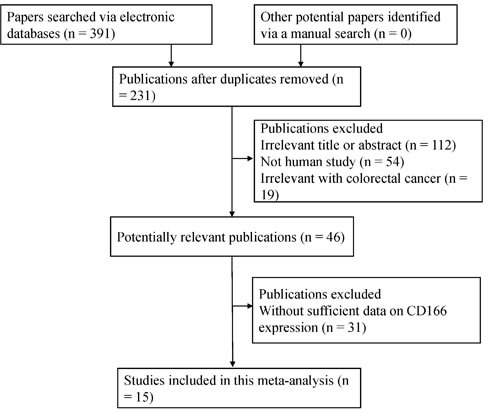
Flow diagram of the selection procedure for this study

Initially, 391 publications were retrieved by the mentioned search strategy. According to the inclusion criteria, 15 eligible studies [18–32] were identified in the final meta-analysis (Figure 1), including 2,810 patients with CRC, 187 patients with colonic adenoma, and 335 controls with normal colonic mucosa. Of these studies, five studies analyzed the relationship of CD166 expression between CRC and normal colonic mucosa. Four studies analyzed the correlation of CD166 expression between CRC and colonic adenomas. Ten studies assessed the relationship between CD166 expression and the clinicopathological features in CRC. Five studies with the original multivariate analysis data analyzed the prognostic and predictive roles of CD166 expression. The general characteristics of the included studies are summarized in Table 1 and Supplementary Table 1.

**Table 1:** Basic characteristics of 15 eligible publications in this study

First author	Country	Ethnicity	Age	Stage	Stainingpatterns	Cut off scores (IHC)	Cancer	Adenoma	Normal tissue	Clinicopathological features	MA-HR			MA-OR
							N (E+ %)	N (E+ %)	N (E+ %)		OS	DFS	PFS	Predictor (survival)
Weichert 2004 [19]	Germany	Caucasians	65	1-4	M	0%	111 (30.6)	NA	NA	NA	Yes	NA	NA	NA
Zhuang 2007 [32]	China	Asians	NA	1-4	M/C	10%	66 (68.2)	NA	66 (0.0)	Yes	NA	NA	NA	NA
Horst 2009 [27]	Germany	Caucasians	67	NA	M	0%	110 (63.6)	NA	NA	Yes	NA	NA	NA	NA
Lugli 2010 [26]	Switzerland	Caucasians	NA	1-4	M	65%	1274 (60.8)	NA	NA	Yes	NA	NA	NA	NA
Chen 2011 [28]	China	Asians	NA	1-4	M/C	5%	69 (49.3)	40 (25.0)	69 (0.0)	Yes	NA	NA	NA	NA
Piscuoglio 2012 [25]	Switzerland	Caucasians	63	NA	M	0	151 (42.4)	87 (32.2)	120 (28.3)	NA	NA	NA	NA	NA
Tachezy 2012 [24]	Germany	Caucasians	65	NA	M	Spots	300 (76.3)	NA	NA	Yes	Yes	NA	NA	NA
Hansen 2013 [23]	USA	Caucasians	NA	2	M	NA	105 (NA)	NA	NA	NA	NA	NA	NA	Yes
Shafaei 2013 [22]	Iran	Caucasians	59	NA	M	50%	121 (34.7)	NA	NA	Yes	NA	NA	NA	NA
Zhang 2013 [29]	China	Asians	65	1-4	M	10%	57 (42.1)	NA	NA	Yes	NA	NA	NA	NA
Zhou 2013 [30]	China	Asians	55	1-4	M/C	10%	120 (55.0)	20 (20.0)	40 (0.0)	Yes	NA	NA	NA	NA
Sim 2014 [18]	Korea	Caucasians	62	3-4	M	NA	112 (NA)	NA	NA	NA	NA	Yes	NA	NA
Zhu 2015 [31]	China	Asians	63	1-4	M/C	0%	102 (60.8)	40 (15.0)	40 (0.0)	Yes	NA	NA	NA	NA
Manhas 2016 [21]	India	Caucasians	NA	1-4	M/C	0%	54 (70.4)	NA	NA	Yes	NA	NA	NA	NA
Ribeiro 2016 [20]	Brazil	Caucasians	65	2-4	M/C	NA	58 (43.1)	NA	NA	NA	Yes	NA	Yes	NA

IHC: immunohistochemistry; NA: not applicable; M: membrane; C: cytoplasm; N: number of total samples; E+: expression positive status; MA: multivariate analysis; OS: overall survival; DFS: disease-free survival; PFS: progression-free survival; OR: odds ratio; HR: hazard ratio.

### CD166 expression in CRC *vs*. control groups

**Figure 2:**
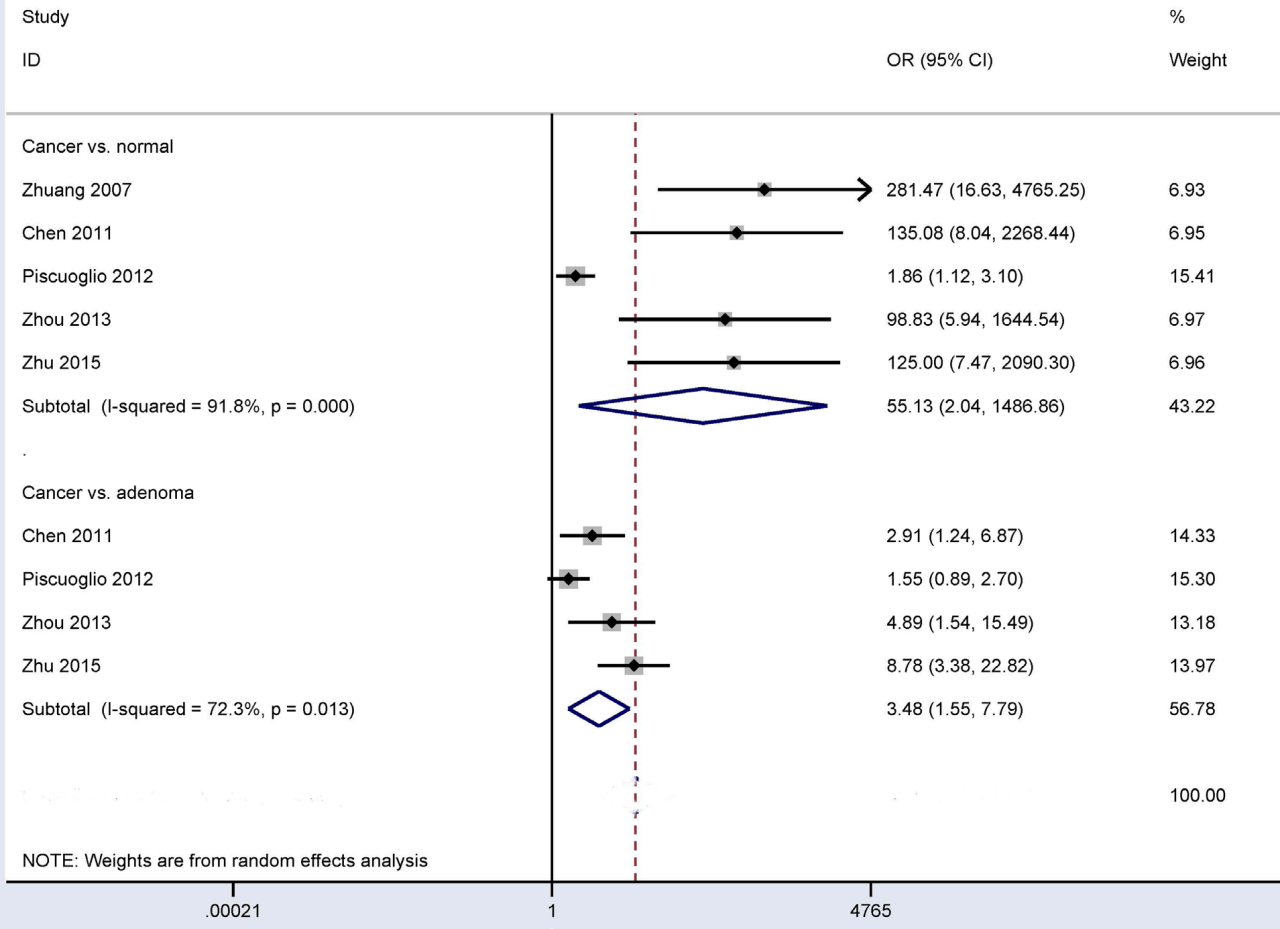
Forest plot of the relationship of CD166 expression between CRC and control groups, cancer *vs*. colonic adenoma OR = 3.48, 95% CI = 1.55-7.79, *P* = 0.002; cancer *vs*. normal colonic mucosa: OR = 55.13, 95% CI = 2.04-1486.86, *P* = 0.017.

The pooled data included five studies with 508 CRC patients and 335 patients with normal colonic mucosa and four studies with 442 CRC patients and 187 adenoma patients (Figure 2). Our results obtained using the random-effects model showed that CD166 expression in CRC had a significantly higher OR than its expression in colonic adenomas or normal colonic mucosa (OR = 3.48, 95% CI = 1.55-7.79, *P* = 0.002 and OR = 55.13, 95% CI = 2.04-1486.86, *P* = 0.017, respectively).

### Associations between CD166 expression and gender and CD166 expression and vascular invasion in CRC

**Figure 3:**
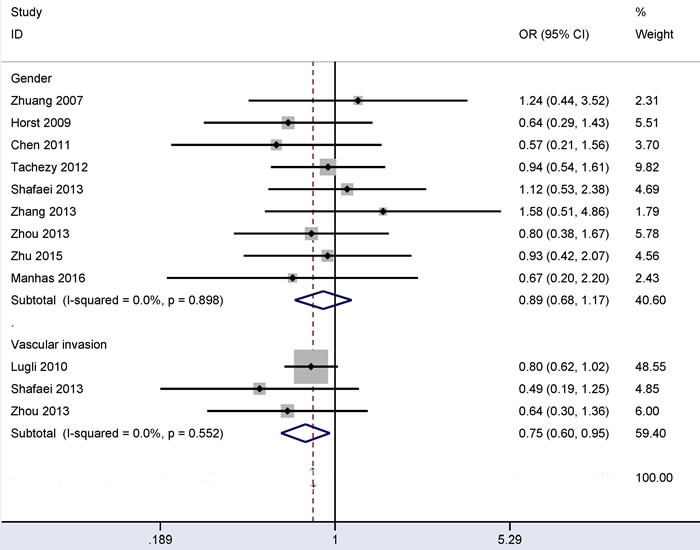
Forest plot of the relationship of CD166 expression with vascular invasion and gender status in colorectal cancer, vascular invasion (yes *vs*. no) OR = 0.75, 95% CI = 0.60-0.95, *P* = 0.017; gender (male *vs*. female): OR = 0.89, 95% CI = 0.68-1.17, *P* = 0.414.

No evidence of heterogeneity was measured in relation to gender or vascular invasion (all *I^2^* = 0.0%) (Figure 3), so the fixed-effects model was applied. The overall OR from three studies, including 411 patients with vascular invasion and 1,075 patients without vascular invasion, showed that CD166 expression was negatively correlated with vascular invasion (OR = 0.75, 95% CI = 0.60-0.95, *P* = 0.017).

The overall OR from nine studies, including 582 male and 417 female patients with CRC, demonstrated that CD166 expression was not correlated with gender (OR = 0.89, 95% CI = 0.68-1.17, *P* = 0.414).

### Associations between CD166 expression and distant metastasis and between CD166 expression and lymph node status in CRC

**Figure 4:**
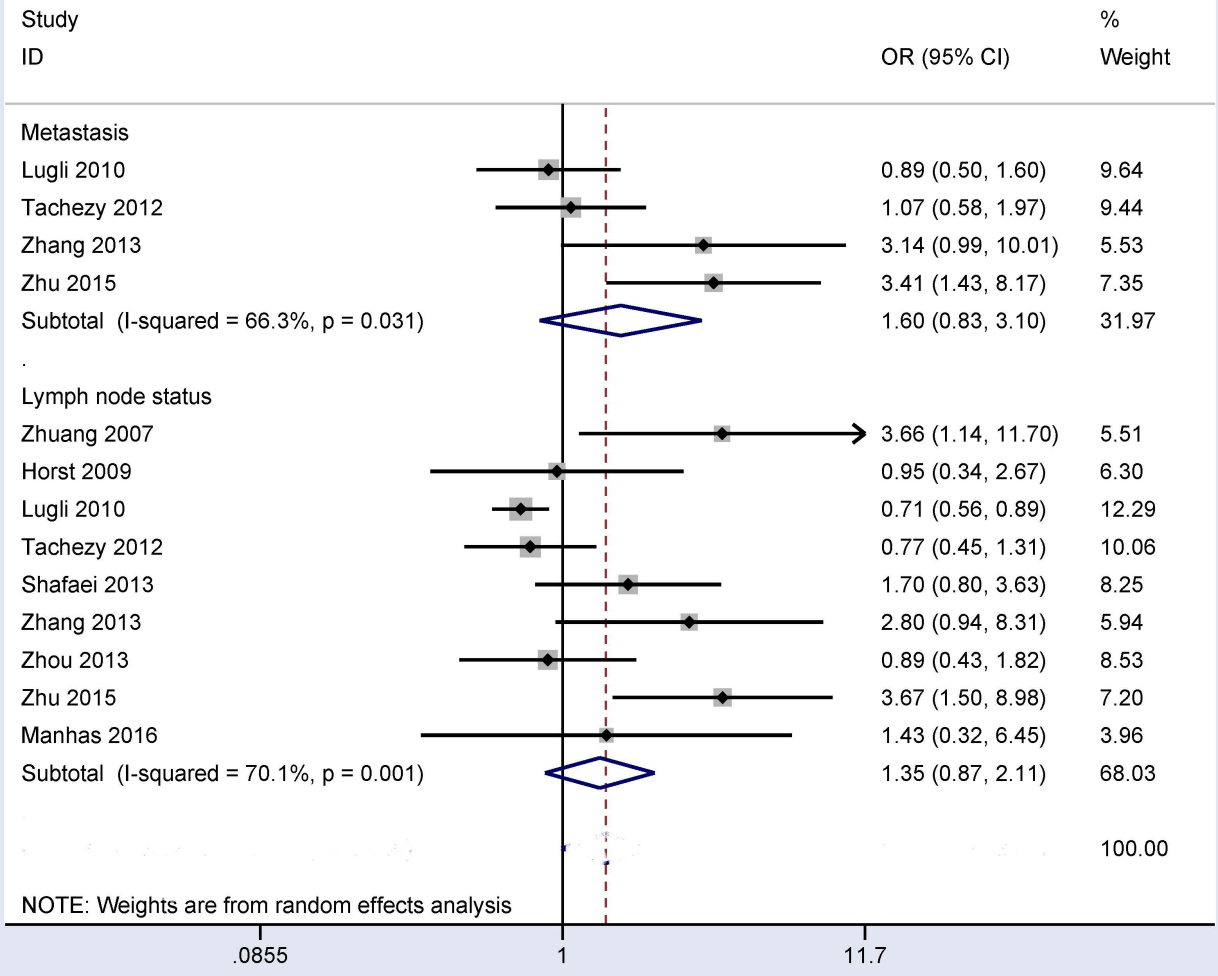
Forest plot of the correlation of CD166 expression with lymph node status and distant metastasis in colorectal cancer, distant metastasis (yes *vs*. no) OR = 1.60, 95% CI = 0.83-3.10, *P* = 0.16; lymph node status (yes *vs*. no): OR = 1.35, 95% CI = 0.87-2.11, *P* = 0.183.

Substantial heterogeneity was detected in relation to distant metastasis and lymph node status (all *I^2^* > 50%), so the random-effects model was used. The results from four studies showed that CD166 expression was not linked to distant metastasis (OR = 1.60, 95% CI = 0.83-3.10, *P* = 0.16) (Figure 4). The results from nine studies showed that CD166 expression was not linked to lymph node status (OR = 1.35, 95% CI = 0.87-2.11, *P* = 0.183) (Figure 4). These data included the comparison of 221 CRC patients with metastasis and 664 CRC patients without metastasis and the comparison of 1,042 CRC patients with lymph node positive status and 1,100 CRC patients with lymph node negative status.

### Associations between CD166 expression and tumor location and between CD166 expression and T classification in CRC

**Figure 5:**
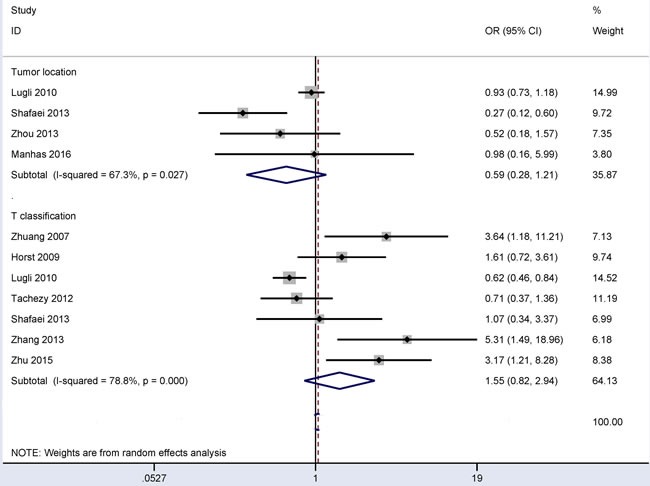
Forest plot of the association of CD166 expression with tumor location and T classification in colorectal cancer, tumor location (left *vs*. right) OR = 0.59, 95% CI = 0.28-1.21, *P* = 0.15; T classification (pT3-4 *vs.* pT1-2): OR = 1.55, 95% CI = 0.82-2.94, *P* = 0.181.

In the comparison of 931 patients with left-sided CRC and 538 patients with right-sided CRC (Figure 5), the results from four studies demonstrated that no significant correlation was observed between CD166 expression and tumor location under the random-effects model (OR = 0.59, 95% CI = 0.28-1.21, *P* = 0.15). In the comparison of 1,577 patients with T3-4 classification and 427 patients with T1-2 classification (Figure 5), the results from seven studies demonstrated that no significant correlation was observed between CD166 expression and T classification under the random-effects model (OR = 1.55, 95% CI = 0.82-2.94, *P* = 0.181).

### Associations between CD166 expression and tumor differentiation and between CD166 expression and clinical stage in CRC

**Figure 6:**
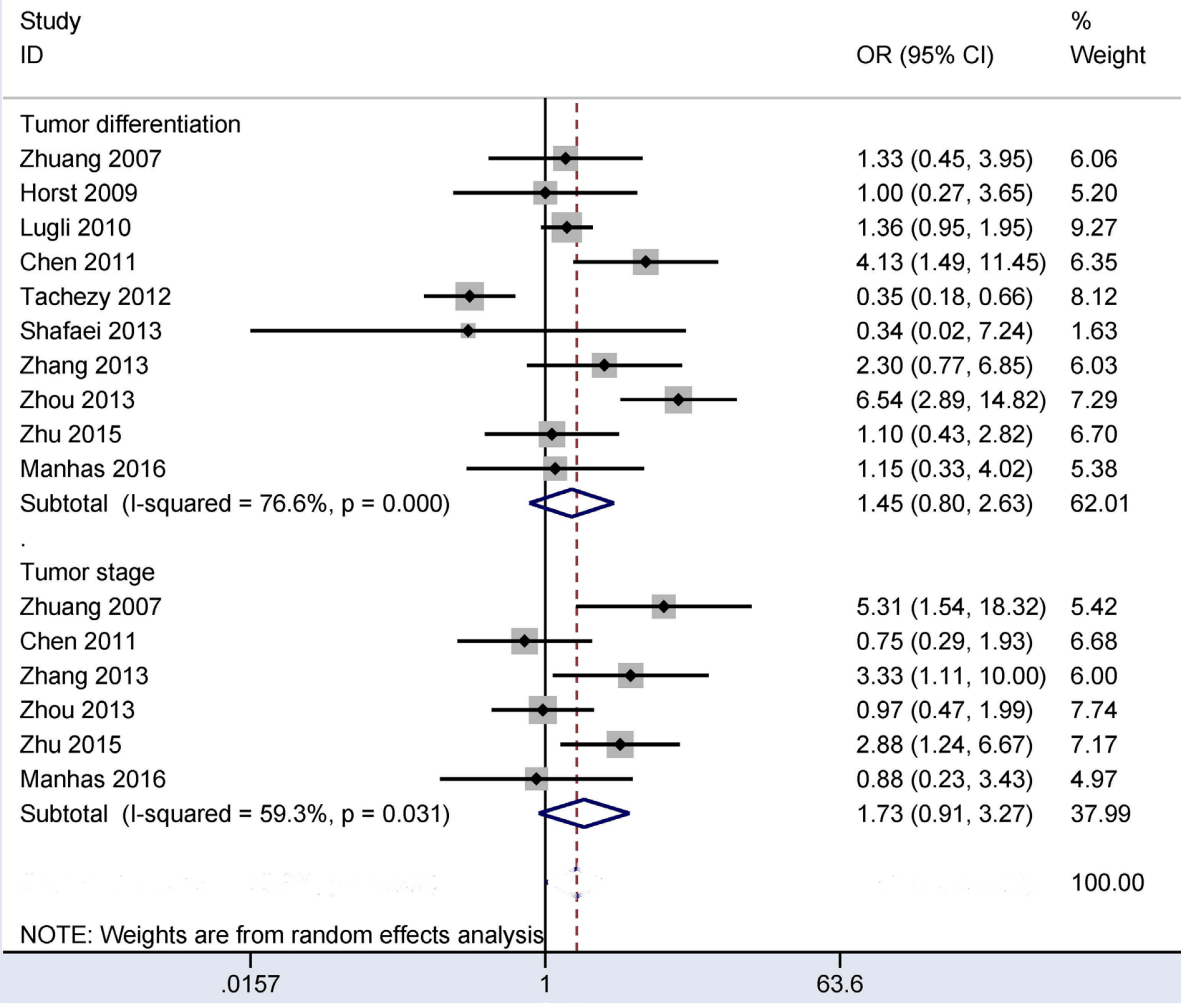
Forest plot of the relationship between CD166 expression and tumor differentiation and clinical stage in colorectal cancer, tumor differentiation (poor *vs.* moderate/well) OR = 1.45, 95% CI = 0.80-2.63, *P* = 0.217; clinical stage (stage 3-4 *vs*. stage 1-2): OR = 1.73, 95% CI = 0.91-3.27, *P* = 0.092.

The random-effects model was applied when there was obvious evidence of heterogeneity in relation to tumor differentiation or clinical stage (all *I^2^* > 50%). When 383 poorly differentiated CRC patients were compared to 1,831 moderately or well differentiated CRC patients, the result from ten studies demonstrated that no significant relationship was found between CD166 expression and tumor differentiation (OR = 1.45, 95% CI = 0.80-2.63, *P* = 0.217) (Figure 6).

The result from six studies with 457 CRC patients showed that no obvious association was found between CD166 expression and clinical stage (OR = 1.73, 95% CI = 0.91-3.27, *P* = 0.092) (Figure 6).

### Prognostic role of CD166 expression in CRC patients using multivariate regression analysis

**Figure 7:**
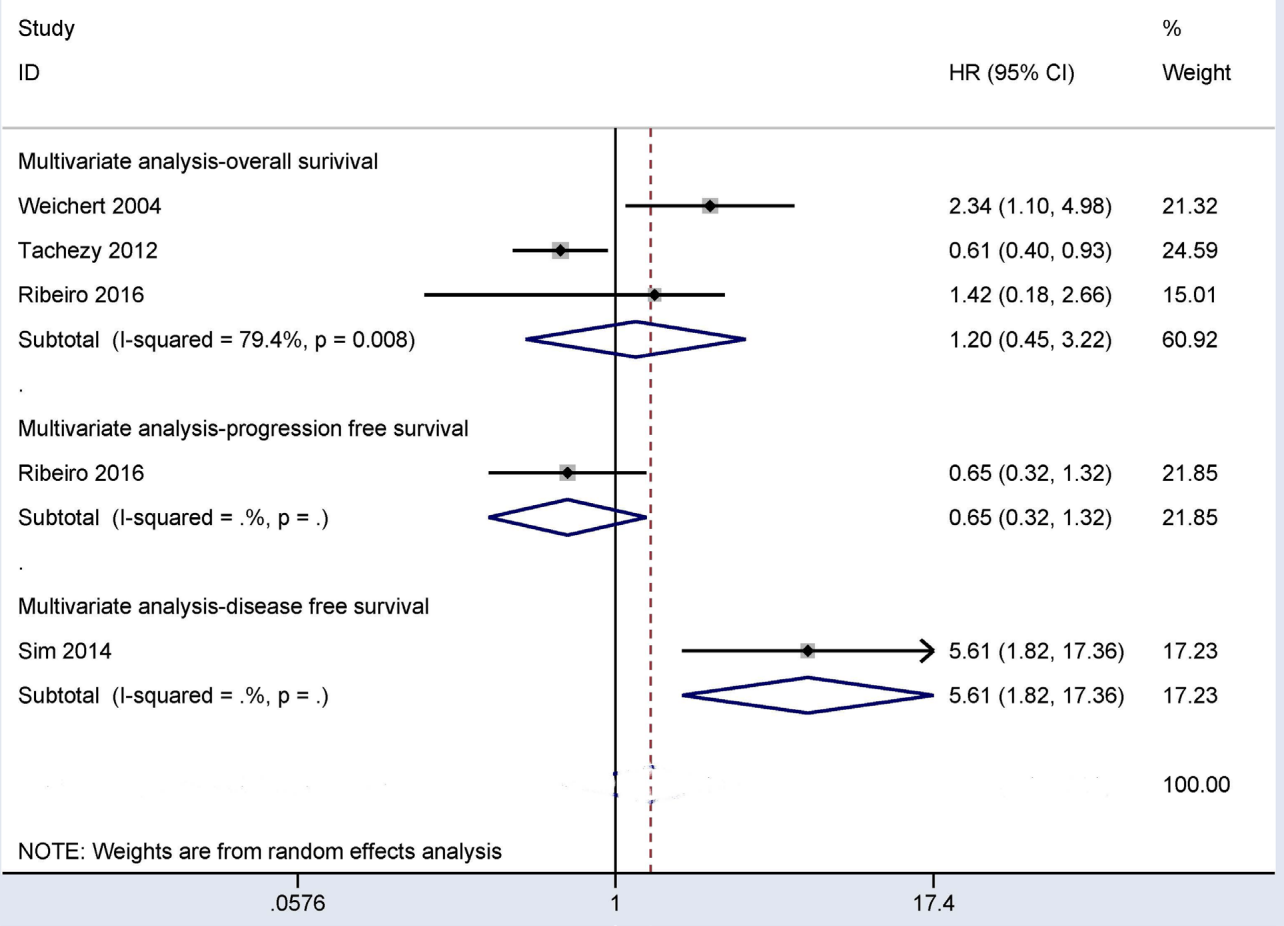
Forest plot showing the prognostic values of CD166 expression in overall survival (HR = 1.20, 95% CI = 0.45-3.22, *P* = 0.72), disease free survival (HR = 5.61, 95% CI = 1.82-17.36, *P* = 0.003), and progression free survival (HR = 0.65, 95% CI = 0.32-1.32, *P* = 0.233) for multivariate analysis

As shown in Figure 7, the results from three studies involving 469 patients with CRC revealed that CD166 expression was not correlated with the overall survival (OS) of patients in multivariate regression analysis (HR = 1.20, 95% CI = 0.45-3.22, *P* = 0.72). One study involving 58 CRC patients reported that CD166 expression was not linked to progression free survival (PFS) in multivariate regression analysis (HR = 0.65, 95% CI = 0.32-1.32, *P* = 0.233). One study including 112 CRC patients reported that a significant association between CD166 expression and disease free survival (DFS) was found in patients with CRC (HR = 5.61, 95% CI = 1.82-17.36, *P* = 0.003) using multivariate regression analysis.

### Predictive role of CD166 expression in CRC patients using multivariate logistic regression analysis

Only one study recorded that CD166 expression may be a predictive marker of clinical outcomes in stage II CRC patients with multivariate logistic regression analysis (OS: OR = 9.97, 95% CI = 1.17-84.90, *P* = 0.035; disease-specific survival: OR = 29.02, 95% CI = 2.17-389.08, *P* = 0.011) [23].

### Publication bias

**Figure 8:**
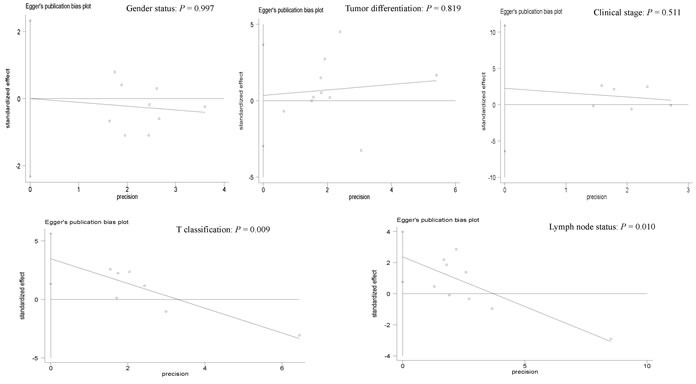
Forest plot of the possible publication bias in relation to gender status (*P* = 0.997), tumor differentiation (*P* = 0.819), clinical stage (*P* = 0.511), T classification (*P* = 0.009) and lymph node status (*P* = 0.010) using Egger's test

Egger's test was applied to detect a possible publication bias in relation to gender, tumor differentiation, clinical stage, T classification or lymph node status (Figure 8). There was obvious evidence of a publication bias in relation to T classification and lymph node status (*P* < 0.05). No evidence of a publication bias was detected in relation to gender, tumor differentiation or clinical stage (*P* > 0.1).

## DISCUSSION

There are a variety of cell surface markers used to identify and enrich CSCs from different human cancers, such as CD44, CD24, CD29, CD133 and epithelial cell adhesion molecule (EpCAM) [33, 34]. As a CSC marker, CD166 expression may be involved in the progression, metastasis and prognosis of several malignant tumors [16, 35, 36]. Immunohistochemical staining of CD166 was found to be frequently expressed in CRC [18, 20, 21], with frequency ranging from 30.6% [19] to 76.3% [24]. CRC generally derives from normal colonic mucosa that turns into adenomas; then these adenomas progress to malignancy through genetic and environment factors [37, 38]. Our findings indicated that CD166 expression was notably higher in CRC than in colonic adenomas or normal colonic mucosa, suggesting that CD166 expression may be related to the carcinogenesis of CRC. We found that the number of cancer and control groups were not well matched, which may have led to the heterogeneous results.

Next, the associations of CD166 expression with clinicopathological characteristics were controversial in CRC. Horst *et al* reported that no significant relationship was found between CD166 expression and clinicopathological features, including gender, lymph node status, T category, and tumor differentiation [27]. Tachezy *et al* found CD166 expression was significantly associated with tumor grade, but not linked to gender, lymph node status and T category [24]. CD166 expression was found to be associated with T category and lymph node status in CRC by Lugli *et al* [26]. Our study in a large series of patients with CRC suggested that CD166 expression was not associated with these clinicopathological features, including gender, tumor location, distant metastasis, lymph node status, clinical stage, T classification or tumor differentiation. Interestingly, CD166 expression was correlated with vascular invasion status. Lugli *et al* reported that CD166 expression showed a trend towards a strong negative association with vascular invasion (*P* = 0.076) in a large population (1,245 CRC patients) [26]. Two other studies with small populations (less than 130 CRC patients per study) reported no correlation between CD166 expression and vascular invasion status (Figure 3). The current meta-analysis involving 1,486 patients with CRC showed that CD166 expression was significantly lower in patients with vascular invasion than in patients without vascular invasion (OR = 0.75, *P* = 0.017). This finding suggested that CD166 expression may significantly decrease the risk of CRC patients with vascular invasion and that CD166 may serve as a potential drug therapy target for patients with vascular invasion.

Finally, the results on the prognostic role of CD166 expression were inconsistent for OS using multivariate regression analysis [19, 20, 24]. The current study involving 469 CRC patients revealed that CD166 expression was not correlated with OS of CRC patients in multivariate regression analysis (HR = 1.20, 95% CI = 0.45-3.22, *P* = 0.72). In addition, Ribeiro *et al* reported that no correlation was observed between CD166 expression and PFS using multivariate regression analysis (HR = 0.65, 95% CI = 0.32-1.32, *P* = 0.233) [20]. The correlation between CD166 expression and DFS was observed using multivariate regression analysis by Sim *et al* (HR = 5.61, *P* = 0.003) [18]. In the future, additional studies with large sample sizes will be needed to validate the prognostic value of CD166 expression in OS, PFS and DFS using multivariate regression analysis.

## LIMITATIONS AND PROSPECTS

Our study had several potential limitations. First, although the PubMed, EMBASE, EBSCO, CNKI, and Wanfang literature databases were systematically searched to minimize any potential publication bias as completely as possible, potential publication bias was observed in relation to T classification and lymph node status. The possible reasons for publication bias are stated as follows: 1) articles with positive conclusions are more easily published than articles with negative conclusions, which are absent; and 2) publications with other styles, such as unpublished papers and conference abstracts, were excluded based on insufficient information. Second, the main population consisted of Caucasian and Asian populations, while the inclusion of other ethnic populations, such as Africans, was limited. Third, the cut-off values of CD166 expression from the eligible studies were different; in the future, CD166 expression should be defined as positive or negative based on a standard. Fourth, the prognostic role of CD166 expression using multivariate regression analysis in OS, PFS and DFS was analyzed in a small population with CRC; additional large-scale studies are necessary to further validate the clinical outcomes of CD166 expression on CRC patients based on larger sample sizes. Finally, only one study in stage II CRC patients reported that CD166 expression may become a predictive biomarker of OS and disease-specific survival using multivariate logistic regression analysis [23], which suggests that additional studies with large populations are needed to confirm the predictive role of CD166 expression in CRC patients with the detailed tumor staging.

## CONCLUSIONS

Our meta-analysis suggested that CD166 expression is associated with the carcinogenesis of CRC and a decreased risk of patients with vascular invasion. CD166 expression may be correlated with a poor prognosis in DFS, and CD166 may become a predictive biomarker of survival for stage II CRC patients. CD166 expression is not correlated with gender, tumor location, distant metastasis, lymph node status, clinical stage, T classification, tumor differentiation, or the prognosis of CRC patients in OS and PFS using multivariate regression analysis. Further large-scale studies with larger sample sizes should be conducted to validate the prognostic and predictive values of CD166 expression in patients with CRC.

## MATERIALS AND METHODS

### Search strategy

The PubMed, EMBASE, EBSCO, CNKI and Wanfang literature databases were searched to obtain the relevant literature published before November 07, 2016. The following key words and terms were used during the search: (CD166 OR ALCAM OR activated leukocyte cell adhesion molecule) AND (colorectal cancer OR colorectal tumor OR colorectal carcinoma OR colorectal neoplasm OR CRC). The references of eligible publications were also scanned to find additional potential studies.

### Selection criteria

The eligible studies in this meta-analysis had to meet the following inclusion criteria: 1) patients with CRC were diagnosed based on histopathological examination; 2) studies regarding CD166 expression were detected using an immunohistochemistry (IHC) method; 3) CD166 expression was defined as positive in the original publications; 4) studies provided sufficient data to estimate the correlation of CD166 expression between CRC and colonic adenomas, and normal colonic mucosa, and in relation to the clinical features of CRC; 5) studies provided the original information from multivariate regression analysis to assess the prognostic or predictive role of CD166 expression in patients with CRC. Only the complete study with the most information was included when authors published more than one paper using overlapping samples.

### Data extraction

All available data were collected from all of studies, including the surname of the first author, year of publication, country, ethnicity, cut-off value (positivity), age, immunohistochemical staining patterns, number of tissue samples, frequency of expression, clinicopathological features, such as gender, tumor location, distant metastasis, lymph node status, vascular invasion, clinical stage, T classification and tumor differentiation, as well as OS, DFS, PFS and disease-specific survival with multivariate analysis. Control groups included colonic adenomas and normal colonic mucosa. Stages ≤ 2 were defined as early stage, and stages of 3-4 were defined as later stage.

### Statistical analysis

Data analyses were conducted using Stata software 12.0 (STATA Corp., College Station, TX, USA). The overall odds ratios (ORs) and their corresponding 95% confidence intervals (95% CIs) were calculated to estimate the correlation of CD166 expression between CRC and control groups, and in relation to patient characteristics (such as gender, tumor location, distant metastasis, lymph node status, vascular invasion, clinical stage, T classification and tumor differentiation.) The combined hazard ratios (HRs) with their 95% CIs were also calculated to analyze the prognostic role of CD166 expression in patients using multivariate analysis. The Cochran's Q statistic and *I²* test were applied to evaluate possible heterogeneity among studies [39]. A random-effects model was used to make the results more reliable for this meta-analysis when there was substantial heterogeneity (*I^2^* > 50% or *P* < 0.1); otherwise, the fixed-effects model was performed [40, 41]. The potential publication bias was detected using Egger's test in cases with more than five studies [42]. A *P* value less than 0.05 was considered statistically significant.

## SUPPLEMENTARY TABLE


