# Supplementary material for: Clinicopathological, prognostic and predictive value of CD166 expression in colorectal cancer: a meta-analysis
Source: Oncotarget. 2017 Apr 26;8(38):64373–84. doi: 10.18632/oncotarget.17442 (PMC5610009; doi:10.18632/oncotarget.17442)
Supplement: Supplementary file 1 [file oncotarget-08-64373-s001.pdf]

Clinicopathological, prognostic and predictive value of CD166 expression in colorectal cancer: a meta-analysis

SUPPLEMENTARY MATERIALS

Supplementary Table 1: General information on CD166 expression with the clinicopathological features of patients with CRC.

First author	Cases	Male	Female	Left	Right	Well/Moderately	Poorly	Stage 1-2	Stage 3-4	T 3-4	T 1-2	Node+	Node -	Vascular invasion +	Vascular invasion -	Metastasis (yes)	Metastasis (no)
		E+/N	E+/N	E+/N	E+/N	E+/N	E+/N	E+/N	E+/N	E+/N	E+/N	E+/N	E+/N	E+/N	E+/N	E+/N	E+/N
Horst 2009	110	38/64	32/46			63/99	7/11			48/71	22/39	54/87	12/19				
Lugli 2010	1274			492/820	272/440	658/1096	100/149			594/1011	165/237	329/582	417/645	194/341	564/904	63/81	275/345
Tachezy 2012	300	135/178	94/122			200/250	29/50			170/227	59/73	114/154	115/146			61/79	168/221
Shafaei 2013	121	23/64	19/57	18/74	23/42	33/89	0/2			37/106	5/15	22/53	20/68	7/30	35/91		
Manhas 2016	54	20/30	18/24	5/7	23/32	25/37	12/17	19/26	12/17			10/13	21/30				
Chen 2011	69	20/45	14/24			14/40	20/29	18/34	16/35								
Zhang 2013	57	17/37	7/20			12/35	12/22	9/31	15/26	20/36	4/21	14/25	10/32			11/18	13/39
Zhou 2013	120	38/72	28/48	14/30	15/24	23/66	42/54	36/65	30/55			31/58	35/62	19/40	47/80		
Zhu 2015	102	33/55	29/47			47/78	15/24	16/36	46/66	53/79	9/23	32/41	30/61			33/43	29/59
Zhuang 2007	66	26/37	19/29			27/41	18/25	20/37	25/29	36/47	9/19	24/29	21/37				

N: number of the total samples; E+: expression positive status; CRC: colorectal cancer.
